# In Vitro Priming of Human T Cells by Dendritic Cells Provides a Screening Tool for Candidate Vaccines for *Burkholderia pseudomallei*

**DOI:** 10.3390/vaccines9080929

**Published:** 2021-08-22

**Authors:** Durga Reddi, Lydia Durant, David Bernardo, Alistair Noble, Nicholas R. English, Philip Hendy, Graeme C. Clark, Joann L. Prior, Ethel Diane Williamson, Stella C. Knight

**Affiliations:** 1 Antigen Presentation Research Group, Imperial Centre for Translational and Experimental Medicine, 72 Du Cane Road, London W12 0NN, UK; dreddi@parkinsons.org.uk (D.R.); l.durant@imperial.ac.uk (L.D.); d.bernardo.ordiz@gmail.com (D.B.); Alistair.Noble@pirbright.ac.uk (A.N.); n.english@imperial.ac.uk (N.R.E.); p.hendy@imperial.ac.uk (P.H.); s.knight@imperial.ac.uk (S.C.K.); 2 Gut Microbes & Health Program, Quadram Institute Bioscience, Norwich NR4 7UQ, UK; 3 St Mark’s Hospital, London North West University Healthcare NHS Trust, Harrow UT 84124, UK; 4 Defence Science and Technology Laboratory, Porton Down SP4 0JQ, UK; gcclark@dstl.gov.uk (G.C.C.); jlprior@dstl.gov.uk (J.L.P.)

**Keywords:** dendritic cells, *Burkholderia pseudomallei*, T cell priming

## Abstract

Murine dendritic cells, when pulsed with heat-killed *Burkholderia pseudomallei* and used to immunise naïve mice, have previously been shown to induce protective immunity *in vivo*. We have now demonstrated the *in vitro* priming of naïve human T cells against heat-killed *B. pseudomallei*, by co-culture with syngeneic *B. pseudomallei-*pulsed dendritic cells. Additionally, we have enriched the DC fraction such that a study of the differential response induced by pulsed DCs of either myeloid or plasmacytoid lineage in syngeneic human T cells was achievable. Whilst both mDCs and pDCs were activated by pulsing, the mDCs contributed the major response to *B. pseudomallei* with the expression of the migration marker CCR7 and a significantly greater secretion of the proinflammatory TNFα and IL1β. When these DC factions were combined and used to prime syngeneic T cells, a significant proliferation was observed in the CD4^+^ fraction. Here, we have achieved human T cell priming *in vitro* with unadjuvanted *B. pseudomallei*, the causative organism of melioidosis, for which there is currently no approved vaccine. We propose that the approach we have taken could be used to screen for the human cellular response to candidate vaccines and formulations, in order to enhance the cell-mediated immunity required to protect against this intracellular pathogen and potentially more broadly against other, difficult-to-treat intracellular pathogens. To date, the polysaccharide capsule of *B. pseudomallei*, fused to a standard carrier protein, e.g., Crm, looks a likely vaccine candidate. Dendritic cells (DCs), providing, as they do, the first line of defence to infection, process and present microbial products to the immune system to direct downstream immune responses. Here, we have sought to use DCs *ex vivo* to identify immunogenic products from heat-killed *B. pseudomallei*. Using practical volumes of fresh human donor blood, we show that heat-killed *B. pseudomallei* activated and stimulated the expression of pro-inflammatory cytokines TNF-α, IL-1β and IL-6 from both myeloid and plasmacytoid DCs. Furthermore, *B. pseudomallei-*pulsed DCs cultured with naïve syngeneic T cells *ex vivo*, induced the activation and proliferation of the CD4^+^ T-cell population, which was identified by cell surface marker staining using flow cytometry. Thus, both DC subsets are important for driving primary T helper cell responses to *B. pseudomallei* in healthy individuals and have the potential to be used to identify immunogenic components of *B. pseudomallei* for future therapies and vaccines.

## 1. Introduction

*Burkholderia pseudomallei* (*B.psm*) is a Gram-negative infectious bacterium and the causative agent of melioidosis, a disease endemic to parts of south-east Asia. Disease may present as a chronic or acute infection of skin, lung, liver or spleen and can lead to pneumonia and septicaemia [1]. After initial exposure, *B.psm* replicates intracellularly within epithelial or phagocytic cells and can evade host immunity [2]. Due to the multiple routes of exposure, active efflux of antibiotics by the bacteria and the lack of an approved vaccine, infection with *B.psm* is problematic to manage. To date, the polysaccharide capsule of *B. pseudomallei*, fused to a standard carrier protein, e.g., Crm, has been pursued as a promising vaccine candidate. In order to eradicate this intracellular infection, it will be essential for melioidosis vaccine candidates to induce effective cellular immunity. Dendritic cells (DCs) have a key role in the induction of cellular immune responses, by processing and presenting microbial products to the immune system to direct downstream immune responses. DCs express pattern recognition receptors (PRR) that recognise the pathogen-associated molecular patterns (PAMPs) within microbes. DCs internalise microbial components by receptor-mediated endocytosis, then process and present antigens associated with major histocompatibility complex (MHC) class II to CD4^+^ or MHC class I to CD8^+^ T cells residing in local lymph-nodes. Upon stimulation, DCs also express CCR7, allowing their migration to local lymph-nodes, and maturation markers including CD80, CD86 and CD40, which provide co-stimulatory signals to T cells. Finally, DCs secrete a variety of cytokines that direct specific T-cell responses. 

The two major subsets of DCs in humans are myeloid and plasmacytoid. Myeloid DCs (mDCs) are typically responsive to bacteria, whereas plasmacytoid DCs (pDCs) respond to viruses [3], although pDCs can be activated by some bacteria including *Staphylococcus aureus* and *Streptococcus pyogenes* [4,5]. Moreover, pDCs co-cultured with mDCs responsive to bacterial stimulation show a unique capacity to mature and be activated, suggesting synergy between the two subsets [6]. Human and murine pDCs internalise and cause the killing of *B.psm* [7]. Furthermore, the passive transfer into mice of bone-marrow (BM)-derived DCs that had been pulsed with heat-killed *B. pm* and matured in the presence of CpG, induced protective immunity against subsequent exposure to *B.psm*, demonstrating the significant role for DCs in driving protective cell-mediated immune responses [8].

The focus of this study was to investigate whether a primary human T-cell response to *B.psm* could be induced *in vitro* by co-culture with syngeneic DC, in order to establish a system to analyse a protective human cell-mediated response to the bacterium. To achieve this and using practical volumes of fresh human donor blood, we have enriched for DCs and pulsed these with heat-killed *B.psm* K96243, the genome-sequenced strain of *B.psm* [1]. A sufficient quantity of DCs were derived to enable the separation of myeloid and plasmacytoid subsets and study of their phenotype (activation and cytokine expression) and function (ability to stimulate primary T-cell responses) after pulsing with *B.psm*. We have found that the major response to unadjuvanted and heat-killed *B.psm* came from the myeloid population. When the total DC faction was pulsed with *B.psm* prior to co-culture with syngeneic naive T cells, a significant proliferation of the gated CD4+ population was observed, together with the detection of T cell activation markers, indicating *in vitro* priming. We propose that this *in vitro* human cell system may be a useful approach for screening candidate vaccines in order to enhance a protective CMI response. Furthermore, our methodology could be used to assess memory responses in *B.psm*-exposed individuals.

## 2. Methods

### 2.1. Bacterial Preparation

*B. pseudomallei* K96243 was cultured in L-broth and on L-agar at 37 °C. Bacterial stocks were maintained at −80 °C as 20% glycerol suspensions. *B. pseudomallei* K96243 was handled at Advisory Committee for Dangerous Pathogens (ACDP) containment level 3. The bacteria were heat-killed as previously reported [8].

### 2.2. Healthy Donors

Blood (30–50 mL) was collected from healthy volunteers with no known autoimmune or inflammatory diseases, allergies or malignancies. Ethical approval was obtained from the Health Research Authority UK and London Brent Research Ethics Committee (05.Q0405.71). Written informed consent was received from participants prior to inclusion. For each independent experiment, a minimum of 3 and a maximum of 6 donors were used, as specified in the Figure legends.

### 2.3. Dendritic Cell Enrichment

Peripheral blood mononuclear cells (PBMCs) were isolated by centrifugation over Ficoll-Paque Plus (Amersham Biosciences, Chalfont St. Giles, UK). Total mDCs and pDCs were negatively enriched from PBMCs using the EasySep™ Human Pan-DC Pre-Enrichment Kit (StemCell Technologies, Cambridge, UK) according to manufacturer’s instructions. Compared to whole PBMCs, where DCs (HLA-DR^+^ and lineage (CD3/CD14/CD16/CD19/CD34)^-^ cells) are between 1–2% of live cells, the purity of DCs (mDCs and pDCs) after enrichment was between 65–70% of live cells. Enriched DCs were cultured in Dutch modified RPMI 1640 (Sigma-Aldrich, Dorset, UK) containing 100 U/mL penicillin/streptomycin, 2 mM L-glutamine, 50 μg/mL gentamicin (Sigma-Aldrich) and 10% foetal calf serum (TCS cell works, Buckingham, UK). 

### 2.4. Bacterial Stimulation

Enriched DCs (5 × 10^4^) were stimulated with ascending concentrations of *B.psm* or medium only (untreated control) for 0, 3, 20 or 24 h at 37 °C and 5% CO_2_. For DCs intracellular cytokine analysis, stimulation with 100 ng/mL of *E. coli* LPS (Sigma-Aldrich, Dorset, UK) was a positive control. 

### 2.5. Cell-Surface Antibody Labelling

After 20-hour incubation, DCs were washed with FACS buffer (1x PBS containing 5% FCS, 1 mM EDTA and 0.02% sodium azide) and labelled with Near-IR Live/Dead Fixable Dead Cell stain (ThermoFischer Scientific) according to kit instructions. Cells were then labelled for 20 min on ice with antibodies, fixed with 1% paraformaldehyde in 0.85% saline and stored at 4 °C until flow cytometric analysis. “Fluorescence minus one” controls were used to determine positive staining for each marker. 

### 2.6. Intracellular Cytokine Analysis

After 3-hour culture, DCs were harvested, labelled with viability stain and surface antibodies as above and fixed and permeabilised using Leucoperm A and B reagents (Bio-Rad, Watford, UK). Cells were labelled intracellularly with antibodies in Leucoperm B. Positive staining for all cytokines was determined by comparing to an untreated control.

### 2.7. Enzyme Linked Immunosorbent Assay (ELISA)

Enriched DCs were cultured with *B.psm* for 24 h and supernatants stored at −20 °C. Concentrations of IL-6, IL-1β and TNF-α were measured using DuoSet ELISA kits (R&D Systems, Abingdon, UK) as per manufacturers’ instructions.

### 2.8. DC: T Cell Co-Cultures

Enriched DCs were stimulated with *B.psm* for 20 h. Total T cells were purified from the same donor’s PBMCs using the EasySep™ Human T cell Isolation kit (StemCell Technologies). Purity of total CD4^+^ and CD8^+^ T cells was estimated at 94%. After 20-hour culture, T cells were labelled with Cell Trace Violet (CTV) dye (Thermo Fisher) as per manufacturer’s instructions. CTV-labelled T cells (2.5 × 10^5^) were co-cultured with either 10% (2.5 × 10^4^) or 5% (1.25 × 10^4^) *B.psm*-pulsed DCs for 7 days. As a positive control, 2.5 × 10^5^ CTV-labelled T cells were stimulated with 5 µg/mL of plate-bound anti-CD3 (300331, Biolegend) and 5 µg/mL of soluble anti-CD28 (302913, Biolegend, London UK). CTV-labelled T cells cultured in medium only were an unstimulated control. 

### 2.9. Flow Cytometry Analysis

Cells were acquired on the FACSCanto II Flow Cytometer (BD Biosciences) and analysed using FlowJo software version 10.

### 2.10. Statistical Analysis

One-way or two-way ANOVA followed by Dunnett’s multiple comparisons test was used to compare experimental groups to medium-only controls. A Student’s paired *t*-test was used to compare T-cell proliferation in the *B.psm* group to untreated control. Statistical analysis used GraphPad Prism version 7.0.

## 3. Results

### 3.1. B. pseudomallei Activates Both mDCs and pDCs Enriched from Fresh Blood 

**Figure 1:**
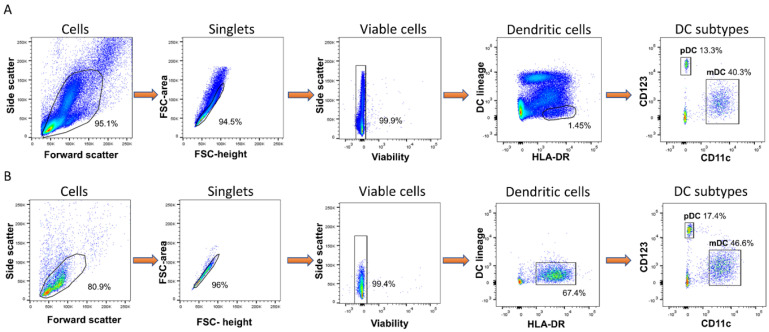
Enrichment of human blood myeloid and plasmacytoid dendritic cells from fresh PBMC. DCs were identified using side/forward scatter properties, doublet discrimination and gating on viable cells. Cells that were HLA-DR^+^ and lineage^–^ (CD3, CD14, CD16, CD19 and CD34) were further divided into mDCs (CD11c^+^CD123^-^) and pDCs (CD11c^-^CD123^+^). (**A**) DC identification within PBMC population before DC enrichment. (**B**) DC identification after enrichment.

Immunisation with heat-killed *B.psm* protects mice against a subsequent pathogen challenge, suggesting that the killing of the bacteria preserves protective motifs and antigens [9]. To define an optimal non-toxic concentration of *B.psm*, DCs derived from fresh naive PBMCs were incubated with ascending concentrations of *B.psm* for 20 h, with a baseline 0 h time-point used for comparison. To enable this and subsequent analyses, DCs were enriched from PBMCs such that, whereas DCs (HLA-DR^+^ and lineage (CD3/CD14/CD16/CD19/CD34)^-^ cells) usually comprise between 1–2% of live cells, the content of DCs (mDCs and pDCs) after enrichment was between 65–70% of live cells (Figure 1A,B).

**Figure 2:**
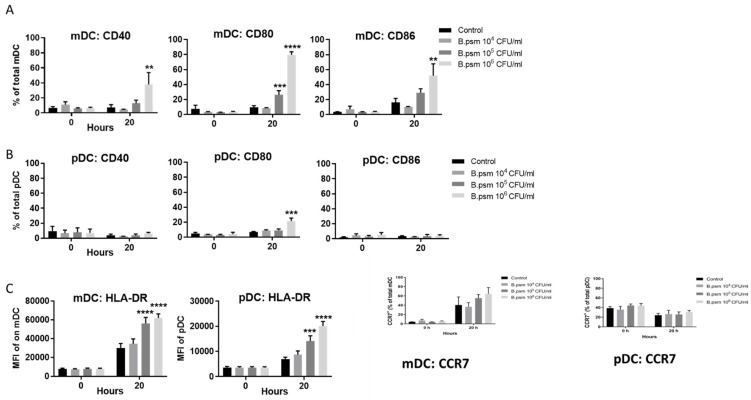
*B. pseudomallei* stimulates maturation and cytokine production by myeloid and plasmacytoid DC in healthy donors. Enriched DCs from healthy donor PBMCs were stimulated for 20 h with *B. pseudomallei* (*B.psm*) and percentage of cells expressing CD80, CD86 and CD40 was measured for (**A**) myeloid (m)DCs and (**B**) plasmacytoid (p)DCs using flow cytometry. (**C**) The median fluorescence intensity (MFI) of HLA-DR on mDCs and pDCs was also determined at 20 h. The expression of lymph-node homing marker CCR7 on mDCs and pDCs subsets was also determined at 20 h. Bars represent mean values ± SEM from three (**A**,**B**) or six (**C**) independent experiments. A two-way (ANOVA) followed by Dunnett’s multiple comparisons test was used to compare experimental groups to the medium only control. ** *p* < 0.01, *** *p* < 0.001, **** *p* < 0.0001.

Using these enriched populations, the expression of activation markers CD80, CD86 and CD40 and lymph node migration marker CCR7 were measured on mDCs and pDCs using flow cytometry. The proportion of mDCs expressing CD80, CD86 and CD40 was significantly increased in response to high concentrations of *B.psm* at 20 h (Figure 2A). The highest concentration of *B.psm* also stimulated a significant increase in the proportion of pDCs expressing CD80, but not CD86 or CD40 (Figure 2B). Whilst the pDCs were less responsive than the mDCs, the median fluorescence intensity (MFI) of HLA-DR (MHCII) on both the mDCs and the pDCs increased significantly after 20 h of *B.psm* incubation (Figure 2C). Further, the proportion of mDCs expressing CCR7 increased in all groups at 20 h, whilst the proportion of pDCs expressing CCR7 was lower at 20 h in all groups; however, these changes were not statistically significant (Figure 2C). Thus, we determined that the highest concentration of 1 × 10^6^ CFU/mL of *B.psm* optimally activated both DC subsets and was used in subsequent experiments.

### 3.2. B.psm Stimulates Proinflammatory DC Cytokine Production

**Figure 3:**
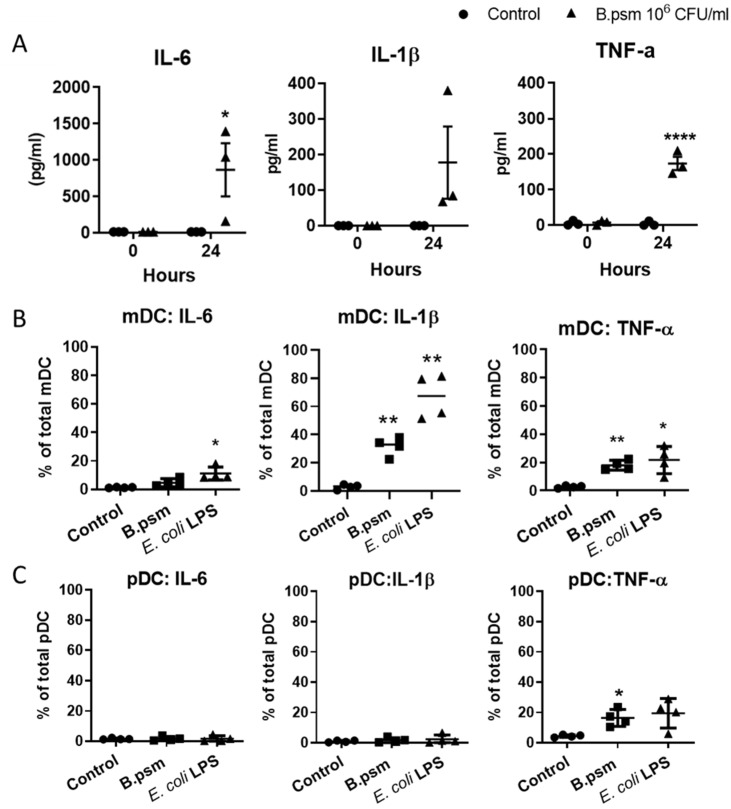
*B. pseudomallei* induces inflammatory cytokine production by myeloid and plasmacytoid DCs in healthy donors. (**A**) Amounts of secreted TNF-α, IL-1β and IL-6 in supernatants of enriched DCs following 24-hour stimulation with *B.psm* or medium only are shown. Percentage of (**B**) mDCs and (**C**) pDCs expressing intracellular IL-6, IL-1β and TNF-α following 3-hour stimulation with *B.psm*, *E. coli* LPS or medium only. MFI of TNF-α in mDCs vs. pDCs after 3-hour stimulation with untreated control, 10^6^ CFU/mL *B.psm* or 100 ng/mL *E. coli* LPS was also determined. Bars represent mean values ± SEM from three (**A**) or four (**B**,**C**) independent experiments. A one-way (ANOVA) followed by a Dunnett’s multiple comparisons test was used to compare experimental groups to the medium only control. * *p*< 0.05, ** *p* < 0.01, **** *p* < 0.0001.

Activated DCs secrete a variety of polarising cytokines that direct protective immune responses. We investigated the production of TNF-α, IL-1β and IL-6 by DCs in response to *B.psm*. At baseline, there was no detectable TNF-α, IL-1β or IL-6 in supernatants of stimulated DCs (Figure 3A). However, after 24 h of pulsing with *B.psm*, TNF-α and IL-6 were significantly increased (Figure 3A). To determine which DC subset was synthesising these cytokines, we stimulated DCs for 3 h with *B.psm* and measured the intracellular cytokines in the mDC and the pDC subsets. *B.psm* stimulation led to a significant increase in the proportion of mDCs expressing TNF-α and IL-1β, while the proportion of mDCs expressing IL-6 was only significantly increased by *E. coli* LPS (Figure 3B). *B.psm* also stimulated a significant increase in the proportion of pDCs expressing TNF-α (Figure 3C). The proportion of mDCs or pDCs expressing TNF-α was similar (approximately 20%), but the MFI of TNF-α was significantly higher in the mDCs compared to the pDCs in response to both *B.psm* (1.78-fold higher) and *E. coli* LPS (1.46-fold higher) stimulation (Figure 3C).

### 3.3. DCs Conditioned with B.psm Stimulate CD4^+^ T-Cell Proliferation

**Figure 4:**
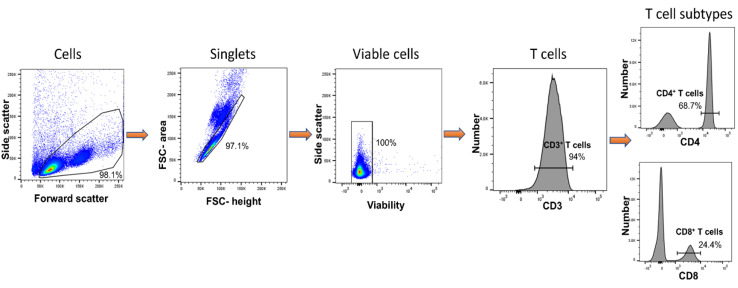
Purification of Total CD4^+^ and CD8^+^ T cells from fresh PBMCs. Total T cells were purified on day 0 from PBMCs and identified using side/forward scatter properties, doublet discrimination and total viable cells. Total CD3^+^ T cells were subdivided using CD4 and CD8 markers to distinguish between CD4^+^ and CD8^+^ T cells, respectively.

**Figure 5:**
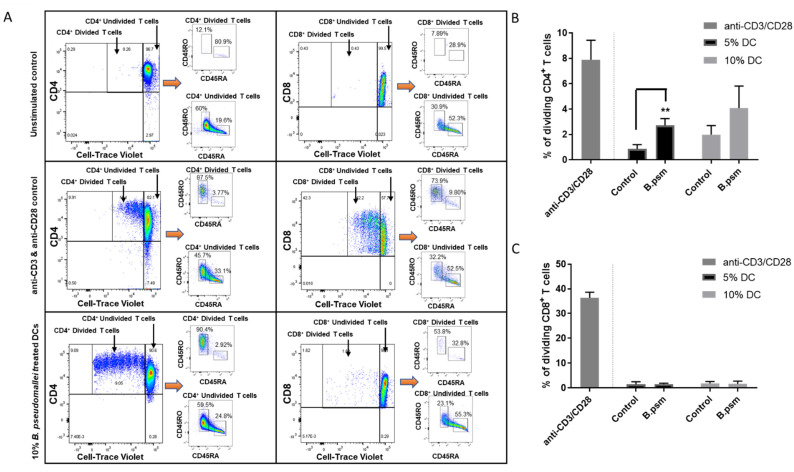
*B. pseudomallei* stimulates a primary CD4^+^ T helper cell response in healthy donors. Enriched DCs were stimulated for 20 h with *B.psm* or medium alone (control). 2.5 × 10^5^ syngeneic total T cells were then co-cultured with either 5% or 10% stimulated DCs for 7 days. Anti-CD3 and anti-CD28 were used as a positive control for T-cell stimulation. (**A**) T cells subsequently acquired on the flow cytometer were divided from the total on the basis of low expression of CTV. These CD4+ CTV^low^ or CD8+ CTV^low^ were proliferated cells and selected for further analysis. Cell activation markers, CD45RA and CD45RO, were used to confirm naïve or activated/memory status, respectively, within the divided and undivided cells. Examples are given of unstimulated (top panels), positive stimulation with anti-CD3 and anti-CD28 (middle panels) or 10% DC-treated with *B.psm* (bottom panels) CD4^+^ and CD8^+^ T cells after 7-days culture. Expression of cell-surface markers CD45RO and CD45RA were used to define activation status of proliferated vs. non-proliferated T cells. Pooled data showing the % of proliferated (**B**) CD4^+^ or (**C**) CD8^+^ T cells after 7 days incubation with 5 or 10% of DCs stimulated with or without *B.psm* or with anti-CD3 and anti-CD28. Bars represent mean values ± SEM from three independent experiments. A Student’s paired *t-*test was used to compare the untreated control to *B.psm* group. ** *p* < 0.01.

*B.psm*-stimulated DCs became activated and produced cytokines; therefore, we next assessed their ability to induce T-cell proliferation. To enable this, we isolated and purified syngeneic T cells *ex vivo* from PBMCs using the flow process depicted in Figure 4, which yielded, using flow cytometry, a CD3+ fraction of 94% purity comprising 68% CD4+ and 24% CD8+ T cells. Enriched DCs (combined mDCs and pDCs) were cultured with *B.psm* for 20 h and added at 5 or 10% to a syngeneic total T-cell population for 7 days. The T cells cultured with anti-CD3+ anti-CD28 were used as a positive control. The T cells subsequently acquired on the flow cytometer were divided from the total on the basis of a low expression of CTV. These CD4+ CTV^low^ or CD8+ CTV^low^ were proliferated cells that were selected for further analysis (Figure 5A). The cell activation markers, CD45RA and CD45RO, were used to confirm the naïve or activated/memory status, respectively, within the divided and undivided cells (Figure 5A). In the left hand panel of Figure 5A, it can be seen that the divided CD4+ T cells activated non-specifically with anti-CD3+ anti-CD28, or specifically activated with *B.psm*-pulsed DC, became predominantly CD45RO+ (87–92% activated) compared with the unstimulated controls; the right hand panels of Figure 5A show that the divided CD8+ T cells could be non-specifically activated (73%) with anti-CD3+ anti-CD28, but were not activated in response to *B.psm*-conditioned DCs. The undivided CD4+ T cells had a much lower activation that did not vary greatly whether they were unstimulated, stimulated with anti-CD3+ anti-CD28 or specifically with *B.psm*-pulsed DCs (60% CD45RO+/19.6% CD45RA; 45.7% CD45RO+/33.1% CD45RA; 59.5% CD45RO+/24.8% CD45RA), respectively; therefore, these responses were not accepted as specific. The undivided CD8+ cells were less than 35% activated in any condition.

The *B.psm*-conditioned DCs added at 5% induced CD4^+^ T-cell proliferation, which was significantly higher than the untreated control after a 7-day culture (Figure 5B). Conversely, the *B.psm-*pulsed DCs added at either 5 or 10% did not significantly induce CD8^+^ T-cell proliferation (Figure 5C). The majority of the CD4^+^ T cells that proliferated in response to *B.psm* treatment were CD45RO^+^, indicating the activation of these cells (Figure 5A). Thus, by enriching for the DC population in human PBMCs, we have achieved an *ex vivo* priming of a CD4+ T-cell response to heat-killed *B.psm*.

## 4. Discussion

We have demonstrated that heat-killed *B.psm* activates both human mDCs and pDCs enriched from fresh PBMCs of healthy individuals and induces their production of pro-inflammatory cytokines TNF-α and IL-1β. While the mDCs were more strongly activated than the pDCs, with a greater expression of costimulatory molecules and IL-1β, both subsets showed a significant up-regulation of MHC-II and produced TNF-α in response to *B.psm*. Furthermore, the combined mDC and pDC fractions stimulated with heat-killed *B.psm* induced a proliferation of syngeneic CD4^+^ T cells *in vitro* to form a small sub-population of memory T cells. This may reflect the *in vivo* situation where the current paradigm is that small populations of memory T cells, which are not long-lived in healthy humans, are maintained by repeated recruitment from the longer-lived naive pool [10], enabling the immune system to have the capacity to respond to a myriad of different antigens and insults.

Our study used modest volumes of blood from single healthy donors to examine the immune response to *B.psm*, which sets it apart from previous studies using bulk buffy coats. We chose to enrich the DCs from PBMCs using a negative selection protocol because, although positive selection gave higher purity, the negatively enriched DCs survived longer in culture. Conveniently, the use of negatively selected DCs allowed us to study both DC subsets simultaneously and build on previous work suggesting that pDCs as well as mDCs are directly involved in the immune response to *B.psm* [7]. Interestingly, both CD4^+^ and CD8^+^ T-cell responses were induced when whole PBMCs from recovered melioidosis patients were cultured with *B.psm* or purified proteins, suggesting that protective memory involves both T-cell types [11]. It will be important in the future to further dissect the specific functions of mDCs and pDCs in promoting protective immune responses to *B.psm* to develop more effective vaccines and therapies. 

DCs have been used in the clinic to treat cancer and autoimmune conditions [12]. More recently, pDCs were used to treat patients with metastatic melanoma by loading them with tumour antigens to induce type I interferon expression [13]. However, extensive clinical use of DCs is restricted by the low numbers of DCs present in peripheral blood and the *ex vivo* loading of DCs with an antigen for vaccine administration is not feasible in terms of cost, labour and the need for tailored therapy for each patient. The targeting of an antigen to DCs *in vivo* using antigen–antibody complexes to DC cell-surface receptors such as CD205 may prove feasible [14]. Live *B.psm* are taken up by DCs and induce the maturation and migration of immature DCs *in vitro* [15]. Although the interaction with the host cell differs for live and killed bacteria, the ability of DCs to become activated by heat-killed *B.psm* and our demonstration that these activated DCs can, in turn, activate even unprimed T cells, underlines the significant role for DCs in directing protective immunity to *B.psm*. By using cells from individuals with suspected melioidosis, it would be possible to apply the methods described herein to test for memory responses not only to killed bacteria, but also to bacterial sub-components, in order to identify those components required for protective immune responses and, thus, develop improved prophylactic strategies.

Furthermore, the methodology described here has the potential to be adopted more widely to screen vaccine candidates for other intracellular pathogens, where promoting a protective cell-mediated immune response is key.

## 5. Conclusions

In conclusion, we have shown that syngeneic CD4^+^ T cells can be primed *ex vivo* to respond with proliferation to *B.psm*-pulsed DC. The protocols used may be exploited to screen for sub-units of *B.psm* able to prime T cells and thus represent potential vaccine sub-units.

## Data Availability

All data relating to this study is reported in the manuscript and figures.

## Author Summary

Melioidosis is an infectious disease endemic to south-east Asia and northern Australia caused by the bacterium *Burkholderia pseudomallei*. Melioidosis presents a significant public health threat because there is no effective vaccine or therapy, leading to a high mortality rate of between 10–50%. We highlight the possibility of immune-based strategies targeting *B. pseudomallei* to better treat and prevent melioidosis. Specifically, we show that dendritic cells—the sentinel cells of the immune system—respond to *B. pseudomallei* in healthy individuals and, in turn, can orchestrate downstream protective immune responses. Thus, dendritic cells may be key players in the development of both vaccines and therapeutics for melioidosis as well as other bacterial-driven diseases.

## Content Includes Material Subject to © Crown Copyright (2021), Dstl

This material is licensed under the terms of the Open Government Licence except where otherwise stated. To view this licence, visit http://www.nationalarchives.gov.uk/doc/open-government-licence/version/3 (acessed on 19 August 2021) or write to the Information Policy Team, The National Archives, Kew, London TW9 4DU, or email: psi@nationalarchives.gov.uk.
